# Supplementary material for: The unbiased estimation of the fraction of variance explained by a model
Source: PLoS Comput Biol. 2021 Aug 4;17(8):e1009212. doi: 10.1371/journal.pcbi.1009212 (PMC8367013; doi:10.1371/journal.pcbi.1009212)
Supplement: Attachment — Submitted filename: response_to_reviewers.docx [file pcbi.1009212.s001.docx]

We thank the reviewers for their critical reading of our manuscript and thoughtful feedback. Below, we repeat the summary provided by the editor and the concerns raised by the reviewers (in blue text) and our response and changes are given thereafter (in black). The locations of changes and important points of emphasis are indicated below with reference to the marked-up version of the manuscript, in which strike-though is used to indicate deletions and underline is used to indicate additions.

**Comments from the Editor:**

The comparisons with the other metrics with real data as suggested by reviewer2 might not only be illustrative but be a good selling point - no?

This is an excellent suggestion. We have now added panel C to Figure 4 to show a comparison of all metrics on the basis of the V4 data’s fit to a DNN. The comparison shows a pattern of estimates that is consistent with our results on simulated data.

I actually believe that some of these other metrics are equivalent to yours mathematically.

None of the other metrics in the manuscript are mathematically equivalent to ours. All estimators, when applied to the same data, give different results. Though the Haefner and Cumming estimator is very close. We have updated the first sentence of the Discussion subsection, ‘Relationship of $\hat{r}^2_{\text{ER}}$ to $\Upsilon$’ to emphasize this:

We have taken a similar approach to Haefner and Cumming [6], commensurately their estimator gives nearly identical results to ours in simulation (less than $<0.0001\%$ power unexplained for SNR=1 vs $>1\%$ for the other estimators), though we provide an important generalization.

But you provide a nice statistical foundation and confidence intervals that I don't believe was presented in prior work (although I did not look at all these papers).

Thank you, that is correct: none of the prior work with competitive performance to our metric (Figure 4A, estimators 5 from the bottom and up) developed confidence intervals.

Also it would be VERY useful to have this code in a notebook published on git. Maybe this is already the case - but as reviewer 2 I did not see that in the manuscript.

This is also an excellent suggestion. We now provide github code to calculate all estimators presented in the paper and confidence intervals. We provide a notebook demonstrating their use on simulated data: <https://github.com/deanpospisil/er_est/blob/main/n2m_examples.ipynb>

**REVIEWER #1**

Main points:

1) At the very base, this correlation correction is based on having a single trial prediction of neural activity. For the mean of this distribution to be meaningful, the model prediction must, in some sense be unimodal (no probabilistic models that might offer two scenarios, i.e., P(neuron not active this trial) vs P(activity | neuron is active this trial)). Such effects can be caused by missed place fields, for example, and have are better explained in terms of population activity. Can a version of this metric still apply to such scenarios? This would be important as the field continues to move in the direction of population activity.

Our work is based on the idea that there is a mean expected response, E(x_i_) to any stimulus x_i_, and in many cases the underlying distribution may be unimodal, but mathematically, it would not have to be. We agree that in cases where the response is believed to reflect a combination of very distinct modes of operation (e.g., stimulus in the RF and stimulus out of the RF, or two discrete states), then some other model, not a single mean rate tuning curve might be better. But even in such cases, one may still be able to ask how well a set of mean responses fits a model. It is beyond the scope of our work to consider the appropriateness of underlying mechanistic models that account for tuning curves, and these remain largely unknown in the cortex.

2) Modeling of neural spiking statistics is an evolving area. The authors introduce a variance-normalized spiking which can handle specific forms of over- and under- dispersion, however I'm curious if the proposed metric can apply to more general statistical models developed over the past decade. For example, Goris et al. 2014 [R1] show a quadratic increase in overdispersion with rate, and follow-up work has further explored more complex relationships in the mean-variance space [R2]. How would the double stochasticity in such models be handeled? The noise is not a clear-cut addition to the numerator and denominator, and so cannot be simply subtracted due to the cross-terms.

We have now tested this, and our tests confirm that the proposed metric can apply to more general statistical models with respect to different mean-to-variance (M-V) relationships. This is because the only requirement of our method is that the mean and variance exist, that the variance is approximately equal across stimuli, and that the central limit theorem can be invoked based on the averaging of independent trials to arrive at the estimated tuning curve. The variance does not need to be the result of additive noise.

The M-V relationships in both papers mentioned by the reviewer can be estimated, and a variance stabilizing transform can be applied. The most popular approach to stabilizing variance is the Box-Cox transform. We now specify this method as a more flexible approach to accounting for diverse M-V relationships in the final paragraph of the Methods subsection ‘Assumptions and terminology for derivation of unbiased estimators’.

Yet, we find empirically that across a variety of M-V relationships, the square root transformation works well. Intuitively, neural spiking is typically restricted in its range, the M-V relationship changes slowly across this range, and thus a linear approximation to the M-V relationship is often good. We demonstrate this by re-running the simulations from Figure 3 except with a variety of non-linear M-V relationships.

Note that M-V relationships in the left panel are all non-linear except where the exponent is equal to 1 (plot is on a log-log scale). Averages of estimator simulations, in the right panel, are color coded to match the M-V relationship to which they were applied. For the estimator \hat{r}^2_ER, these traces overlap because they all give approximately the same average result.

3) I find the discussion on SNR on page 12 a little confusing (Par. starting on line 364). It's not always possible to increase the bin size as there are limiting factors on the chang in the underlying homogeneous spike rate. At some point the rate then becomes meaningless, i.e., you get a sinple "tuning curve" with no time information on the response. This is especially going against the increased emphasis on neural dynamics in the population level that is becoming prominant.

In the 3rd paragraph of our Results sections, ‘Signal-to-noise ratio as recording quality metric’, we now clarify that it may not always be possible to widen the spike counting window and that our normalization on the basis of the counting window is performed simply to allow for comparison across datasets, removing time as a factor.

In the case of spiking neurons, SNR can be improved by increasing the stimulus duration and thus the spike counting window. Under the generally optimistic assumption that spike rate stays constant in the counting window, and assuming that the spike counting window could be changed given experimental constraints, we can normalize $\widehat{\text{SNR}}$ across the data sets to what the $\widehat{\text{SNR}}$ would have been had all spike count windows been 1 second long (Figure~12B). Under these assumptions, this normalization allows us to examine SNR differences across studies removing the counting window length as a factor.

We note that our estimator is not meant to be applied to mean spike counts in bins across time, because the raw counts tend to be correlated. An extension to our estimator would be needed to account for correlations between adjacent time bins. In this paper we assume spike counts at points along the estimated tuning curve are not correlated (as expected when stimuli are randomly interleaved).

In future work we plan to extend our estimator to the case of adjacent time bins. We now emphasize these limitations and extensions in the 2^nd^ paragraph of the Discussion subsection ‘Further work’.

4) The calcium fluorescence experiments requires more detail as to how the model "mean" changes. It has been observed both in real data [R3] and in simulation [R4] that single spikes sometimes do not cause significant changes in the DF/F signal. This is a skewed version of the spike count, and so is the idea here that there is a different statisitcal model for the data that no longer relies on the count of firing events? For example, a linear model on the DF/F changes is possible, but so is a traditional tuning curve model with a conditional probabilistic model over DF/F [R5]. I think the authors mean the former, but this should be clarified mathematically as to what statistical model of "neural firing" is used.

Our method does not depend on responses being firing rates - it generally applies to any set of independent measurements with a mean and a variance. In the first paragraph of the Results subsection, ‘Bias of $\hat{r}^2$ and its correction’, we now clarify that the estimator is designed to be an unbiased estimate between the model and the expectation of any neural signal.

Consider a typical scenario in sensory neuroscience where the responses of a neuron to $m$ stimuli across $n$ repeated trials of each stimulus have been collected and the average of these responses, the estimated tuning curve (Figure~1, dashed green line), is compared to responses predicted by a model (red line). {These responses could be spike counts from a neuron but could just as well be any other neural signal}.

In analyzing the Ca^2+^ responses, we do not consider inference of spiking data from calcium data, because our method can operate directly on the Ca^2+^ signals and such questions are outside the scope of our paper.

5) The demonstration of inconsistancy in r^2 and consistancy in r_ER^2 don't give much of a window into the nonasyptotic biases. For example, while demonstrating how r^2 is bias even in the limit is interesting, it would be interesting to understand as a function of m how fast r_ER^2 converges. Figure 3 seems to indicate a very slow (linear or sublinear?) convergence rate. Is there any way around this given that typical experiments might not have m>1000 stimuli?

Beginning on line 630, we work out the source of the bias in \hat{r}^2_ER but it is not immediately clear how to analytically evaluate this bias. Thus, we rely on simulations to show the bias. We now mention in the final sentence of the first paragraph of the Discussion subsection ‘Further work’ that future work could attempt to provide analytic results on small sample bias of \hat{r]^2_ER:

In addition, analytic results on how this small sample bias varies as a function of critical parameters $m$, $n$ and SNR would be helpful in its interpretation.

To our knowledge, there is no current estimator that will improve this convergence for a data set with a given number of stimuli, repeats, and SNR. Despite this bias, our method is the least biased method we could find. We provide one potential avenue to reduce the low SNR bias in the first paragraph of the Discussion subsection, ‘Further work’.

**REVIEWER #2**

Major concerns

1. (p.4) The assumption that variance is constant, or that the data can be readily scaled for variance to become constant, seems reasonable, but it is important to back up this claim with simulations. One becomes particularly concerned in the case of datasets with very sparse spiking activity and many zero responses, which is typical of neural data. This concern could be addressed with a simulation along the lines of Figs. 2-3 but using more realistic spike-like data mimicking responses to a natural or other complex stimulus.

We agree that the statistical properties of low firing rate responses can be challenging. To address this, we examined simulated spiking data where we held SNR constant at 1 and tested a low and high average spike count (1.5 and 17, blue and orange tuning curves below, left panel), roughly matching the range seen for V4 neuronal responses to natural images (m = 40 stimuli, n = 5 repeats). We found that there was little difference in the bias but that there was an increase in the variability of the estimator when the average spike count was low (right panel, below).

As firing rates become especially low, the biases introduced by low SNR become an issue, but our manuscript already emphasizes the limitations of analyzing data with very low SNR.

We now mention in the second to last sentence of the Results subsection ‘Validation of $\hat{r}^2_{\text{ER}}$ by simulation’ that:

We ran similar simulations on the square root of Poisson distributed spikes counts and found comparable results for both low and high average firing rates.

2. The comparison between methods (Fig. 4) is compelling, but it seems important to perform a similar comparison on a real dataset. Do some of the problematic metrics (CC_norm-pb_, r2_norm-split-sb_) show consistently biased results for the real data? Of course, there is no ground truth here, but it should be possible to show if their estimates are consistently different from r_ER.

This is an excellent suggestion. We have now included a third panel (panel C) in Figure 4 comparing all metrics on the basis of the fit of real V4 data to a DNN model. We find that the basic pattern of biases is reproduced when the estimators are applied to the same data as we mention in the second to last sentence of the final paragraph of the Results subsection, ‘Comparison to prior methods’:

We applied these estimators to neural data from V4 fit by a deep neural network (see Methods, "Electrophysiological data") and found a similar pattern of results where the top six estimators give similar estimates to each other, $\hat{r}^2 (1-\frac{SE^2}{SS_{total}})$ and $\hat{r}^2_{norm-split-SB}$ tend to be greater than these estimators, and the estimators $\hat{r}^2$ and $\text{CC}^2_{\text{norm-PB}}$ are lower (Figure~4C).

3. Can code for measuring r_ER and associated confidence intervals be made available? If it was mentioned somewhere in the manuscript, apologies for missing it. While not absolutely necessary, a simple package with a brief tutorial demonstrating use of this metric would be very helpful to readers and go a long way toward getting researchers to adopt the method.

Thank you for making this important point. We now provide code to calculate all estimators presented in the paper and confidence intervals. We provide a notebook demonstrating their use on simulated data. We link to this code in the new Methods subsection, ‘Software accessibility’: https://github.com/deanpospisil/er_est/blob/main/n2m_examples.ipynb.

Lesser concerns

L67 / L473. The concept of neuronal SNR as a metric for screening responsive vs. non-responsive neurons is not new. It may be that the current study has developed more rigorous approach to assessing responsiveness with an SNR metric. But many, many studies exclude a subset of neurons based on some SNR metric for the reasons highlighted in this ms.

We now emphasize the difference between our SNR metric and the naive method of estimating SNR. The main difference being that a naive estimate of SNR (the ratio of the sample variance across stimulus means to that within stimuli) will positively bias the SNR estimate proportional to \sigma^2/n (trial-to-trial variability divided by number of trials). Whereas our corrected estimator is constructed to remove this bias. We now emphasize in the first paragraph of the Methods subsection ‘Signal-to-noise ratio as recording quality metric’ that:

The removal of this bias in our SNR metric allows for direct comparisons between studies with different numbers of repeats and amounts of trial-to-trial variability.

L176. “cc2_norm” should this be “cc2_norm-split” to match the label in Fig. 4?

We have corrected this typo.

L247. Unclear what is different here, exactly.

We now clarify the improvement in performance:

We employed $\text{BC}_a$ with our parametric bootstrap and found that performance improved relative to the parametric bootstrap (Figure~6 green trace closer to desired $\alpha$ than red for low $r^2_{\text{ER}}$) but still deviated from the desired $\alpha$ for low and high $r^2_{\text{ER}}$.

L269. “typical” Is this the appropriate term here? This unit seems unusually well-described by the sinusoidal model.

We find that most neurons with good SNR (SNR>3.5) are well fit to a sinusoid model (Figure 8, most points left of 3.5 SNR greater than r^2_ER=0.75). We now remove the word typical because it is an unnecessary qualitative statement.

L468. “variance explained for a linear model” versus “Pearson’s r2”. It’s not clear what the difference is between these two scenarios. Perhaps it could be spelled out in a bit more detail?

We now clarify the difference between these two metrics in the second paragraph of the Discussion subsection, ‘Relationship of $\hat{r}^2_{\text{ER}}$ to $\Upsilon$’.

Haefner and Cumming's estimator is more general than the $\hat{r}^2_{\text{ER}}$ we have presented~~: their $\Upsilon$ estimates variance explained for a linear model, ours estimates Pearson's $r^2$~~. The estimate $\hat{r}^2_{\text{ER}}$ measures the variance of the mean centered data explained by a single covariate, and $\Upsilon$ measures the variance explained by a linear combination of up to $m$ covariates. In particular, $\Upsilon$, accounts for the decrease in degrees of freedom available to the noise as more covariates are added. ~~Ours can be seen as a special case of theirs, being identical when estimating the fit of a linear model with a slope and intercept~~. We also provide the more general version of our estimator for the case of variance explained by a linear model (Eqn.~\ref{hat_r2er_cd}) with the advantage discussed in the previous paragraph.

The methods are quite comprehensive in their scope. While quite dense, they appear to have been developed and described quite rigorously.
